# Humor in Workplace Leadership: A Systematic Search Scoping Review

**DOI:** 10.3389/fpsyg.2021.610795

**Published:** 2021-07-27

**Authors:** Caroline Rosenberg, Arlene Walker, Michael Leiter, Joe Graffam

**Affiliations:** School of Psychology, Faculty of Health, Deakin University, Geelong, VIC, Australia

**Keywords:** humor, leadership, workplace, communication, cultural context

## Abstract

Humor studies are increasingly prevalent in workplace and leadership domains, it has shown significant development in the last 40 years. The multifaceted nature of humor means varied definitions and diverse measurement approaches have been approved. As a result, research methodologies and findings are not easily clarified, and have not been synthesized. The aim of this scoping review was to review the existing body of literature relevant to humor in workplace leadership to identify key research areas, methodologies used, guiding theoretical frameworks, and gaps that are persisting over the last 40 years. Using qualitative review methods, four key themes in the research emerged relating to: (1) humor styles and outcomes; (2) humor as communication and discursive resource; (3) variables in the humor and leadership relationship; and (4) cultural context. This review demonstrates significant research progress on the topic of humor in workplace leadership. Research progress and gaps are discussed based on five key questions. Future research directions are outlined and discussed.

## Introduction

*“A little humor is good for the soul”*

Richard Branson

*“A sense of humor is part of the art of leadership, of getting along with people, of getting things done.”*

Dwight Eisenhower

Humor studies are well established in disciplines such as linguistics and social science (Robert and Yan, 2007; Wijewardena et al., 2017). Since Malone (1980) postulated the cases for and against humor in the workplace, a growing body of literature started to emerge in business management, leadership and organizational psychology (Decker and Rotondo, 1999; Scheel and Gockel, 2017). These studies indicate the positive influence humor has on a range of desirable organizational outcomes, such as group cohesiveness (Holmes and Marra, 2006), team performance (Mao et al., 2017), employee resilience and coping (Vetter and Gockel, 2016), citizenship behaviors (Tremblay and Gibson, 2016; Tremblay, 2017), and leadership effectiveness (Mesmer-Magnus et al., 2012). In particular, humor in leadership has attracted an increased amount of empirical research in recent years (Cooper et al., 2018; Kong et al., 2019), as leadership is a key element of organizational effectiveness and business success. Given its social and commercial value, leader traits, leadership competencies and leadership styles have been extensively studied to enhance understanding of the core makeup of a good leader and what constitutes effective leadership (Avolio, 2011). Anecdotally, humor has long been recognized as a desirable trait for leaders and an effective leadership tool that is underutilized (Malone, 1980; Duncan, 1982; Vecchio et al., 2009). However, the challenge of studying humor in leadership begins with the definition of humor being fluid and as a result, it is difficult to assess the construct directly.

Eisenhower was a leader of military and political arenas, yet his quote is recognized in both the popular and academic literature (Bacharach, 2013). From a leadership perspective, appropriate humor use may help leaders to project confidence and competence (RISE, 2016; Bitterly et al., 2017). A Robert Half (2017) survey found that 84% of executives believe that people with a good sense of humor do a better job. From a leadership outcomes perspective, humor can relieve tension, build trust, boost morale, facilitate leaders to build a better relationship with others at work, foster a positive workplace culture, and increase productivity (Holmes and Marra, 2006; Smith, 2013). Investigating the relationship between humor and leadership may help to identify the positive effects and elicit the conditions whereby humor may detract from leadership effectiveness (Buchanan, 2018). Early researchers and popular authors have cautioned that humor is a double-edged sword (Malone, 1980), and that its effect varies in different situations (Duncan, 1982). While researchers have acknowledged the need for further research to understand how humor and leadership interact, empirical evidence remains limited (Vecchio et al., 2009; Wang et al., 2017).

The outcomes and effects of leadership should be reviewed with a contextual lens (Hiller et al., 2011). Leadership in military organizations, sport clubs, leadership for political or social causes are likely to produce different effects and outcomes to leadership in traditional work organizations due to the differences in contextual demands (Goh, 2009; Liden and Antonakis, 2009; Larsson and Vinberg, 2010; Barling, 2014; Ospina, 2017). In order to preserve the internal consistencies of this review, the scope of leadership is limited to workplace leadership, and the specific inclusion and exclusion criteria are discussed in “Methods” section.

There is a recent review on leader humor by Kong et al. (2019). The meta-analysis reviewed 34 quantitative studies, the authors compared the effect of leader trait humor vs. leader humor expression on a range of follower outcome variables. It was found that leader humor expression has a stronger association with follower outcomes compared with leader trait humor. However, trait vs. behavioral humor in leaders represents only one specific area of humor in leadership research, there are many other themes of studies, which will be outlined in the present scoping review. An earlier meta-analysis of positive humor in the workplace (Mesmer-Magnus et al., 2012) was also identified. The authors reviewed 49 studies of positive humor use in the workplace and found that a positive sense of humor is associated with good physical and mental health, buffers workplace stress and promotes effective workplace functioning. It did not specifically focus on humor in leadership, or the negative aspects of humor.

The rationale for this review is to provide a more comprehensive overview of the research landscape, map the key concepts studied, main themes of evidence available on the topic of humor in workplace leadership, which is currently unavailable in the literature. As a result, a scoping review is adopted over a systematic review and meta-analysis. Similar to systematic reviews, scoping reviews follow a rigorous, systematic literature search and identification process; however, unlike systematic reviews, scoping reviews do not analyze data to answer a narrow research question, instead they are exploratory in nature (Colquhoun et al., 2014; Lockwood and Tricco, 2020), and can be used to identify specific research questions for future systematic reviews (Pham et al., 2014). A scoping review is particularly relevant to the topic of humor in workplace leadership, because the topic is heterogeneous, and has not been extensively reviewed. Both quantitative and qualitative studies, as well as theoretical papers are included in the review. The specific aim of this scoping review was to systematically search the literature on “humor in workplace leadership,” to:

1. Map the key concepts and methodologies used in studying the topic, the themes of evidence, guiding theoretical frameworks and propositions.
2. Review and synthesize progress made in the last 40 years, map out the key progresses and identify challenges and gaps that still persist.
3. Make recommendations for future research directions and specific research questions that can be used to guide future systematic reviews.

## Methods

This scoping review adopted the framework developed by Arksey and O’Malley (2005) for systematic search and reporting. The framework outlines an iterative five-stage process of: (1) identifying the research question, (2) identifying relevant studies, (3) study selection, (4) charting the data, (5) collating, summarizing and reporting the results. This review also incorporated several recommendations proposed by Levac et al. (2010) that aimed to clarify and enhance the methodology. Specifically, the stage five recommendation was adopted whereby the process of reporting results was further broken down into three steps: analysis, reporting the results, and considering the meaning of the findings as they relate to the overall study purpose. The results were analyzed and synthesized using Braun and Clarke (2006)’s thematic analysis approach to formulate themes and sub-themes of the current literature. The method is described here in terms of the Arksey and O’Malley (2005) framework.

### Step 1: Identifying the Research Question

The research questions were constructed based on the aims of this review, specifically:

1. What topics have been the focus of empirical research in relation to humor and workplace leadership?
2. What has previously been established in relation to the function and effect of humor in workplace leadership?
3. What challenges and gaps exist in current humor and workplace leadership research and what are the key priorities that should guide future studies?

### Step 2: Identifying Relevant Studies

A preliminary search strategy was developed to identify peer-reviewed studies related to humor in workplace leadership. Two key concepts, humor and leadership were initially used as search terms and trialed with the PsycINFO database, the largest electronic database of peer-reviewed literature in behavioral science and mental health. The search results were then used to further develop and refine the search strategy, including the use of Boolean searching techniques, a title and abstract search, and thesaurus terms used in the database. Workplace was identified as a third key concept and “Work^∗^” was used in a further search strategy to contain the review to workplace settings. However, this significantly reduced the number of identified papers. Following a discussion among the research team it was agreed that the original two key concepts (humor and leadership) should be used in the search strategy and that during the study selection stage, articles that were not workplace related would be excluded. A description of the final search strategy applied to the PsycINFO database and the number of identified articles at each stage of the search are detailed in Table 1.

**TABLE 1:** PsycINFO database search strategy.

Step	Search terms	Results
S1	TI humor OR AB humor	8,359
S2	TI wit OR AB wit	1,059
S3	TI funny OR AB funny	947
S4	DE humor	4,686
S5	S1 OR S2 OR S3 OR S4	10,324
S6	TI leader* OR AB leader*	95,873
S7	DE leadership	36,674
S8	S6 OR S7	99,592
S9	S5 AND S8	279

TI, title search; AB, abstract search; DE, thesaurus search of indexed terms. Both humor and humor were used in the search but returned the same results. Leader* indicates a wild card search for ‘leader’ terms (e.g., leadership).

This search strategy was then applied to three additional relevant databases: Business Source Complete, Cumulative Index to Nursing and Allied Health Literature (CINAHL) Complete, and Psychology and Behavioral Sciences Collection. The search results for each database are listed in Table 2. The search process concluded at the end of May 2020, articles that are published or indexed post May 2020 are not included in this scoping review.

**TABLE 2:** Databases search results.

Databases	Results
Business source complete	458
CINAHL complete	78
PsycINFO	279
Psychology and behavioral sciences collection	33
Total	818

Gray literature was excluded in the search for this review because the intent was to restrict the review to peer-reviewed, academic papers. In addition, an initial search of the gray literature indicated a vast amount of material available and in diverse forms, such as videos, periodicals and blogs. As a result, the feasibility of the study was prioritized (Levac et al., 2010), to focus on the peer reviewed academic literature only. A hand search of the reference lists of the identified articles was carried out progressively during the study screening and selection process.

### Step 3: Study Selection

The study selection process included a recommendation by Levac et al. (2010), that this stage should be considered an iterative process. The study inclusion and exclusion criteria presented in Table 3 were developed as the titles and abstracts were reviewed, and further refined as full articles were reviewed.

**TABLE 3:** Inclusion and exclusion criteria for article selection.

Criterion	Inclusion	Exclusion
Type of publication	Peer-reviewed journal articles	Tributes, in memoriam, or obituaries; editorials, magazine inserts, book reviews, or any gray literature
Language	English	Non-English
Research		
Time period	Not specified for journal articles and dissertations	None
Literature focus	Articles with both “humor” and “leadership” as main concepts and conducted in a workplace setting	Articles that did not focus on either concept; or Articles focused on only one of the two key concepts; or Context not a typical workplace (e.g., school, college, or military unit)
Participants	Members of an organization, including corporate, NFP or government entities	Students (primary, secondary or tertiary), patients, military cadets, athletes from a sports club

A scan of the titles following the search revealed that a large number of identified articles were tributes, in memoriam, or obituaries, and these were excluded along with duplicates in the initial study selection process. In addition, non-English language articles were excluded. A review of the abstracts found that some studies had been conducted in schools or colleges investigating how humor effects lectures. These studies were excluded based on the context not being a typical workplace and the research focus not relating to leadership.

Following full-text review of the remaining articles, it was apparent that a large proportion of articles were either missing leadership or humor as key concepts. For example, some articles focused on humor and general team dynamics or group effectiveness in the workplace, rather than leadership specifically. Other articles focused on laughter, teasing, or ostracizing, which are related to humor, but only represent one aspect of humor. Articles that did not have both leadership and humor as components of the research were excluded from this review. Table 3 summarizes the complete inclusion and exclusion criteria used in this review.

**FIGURE 1:**
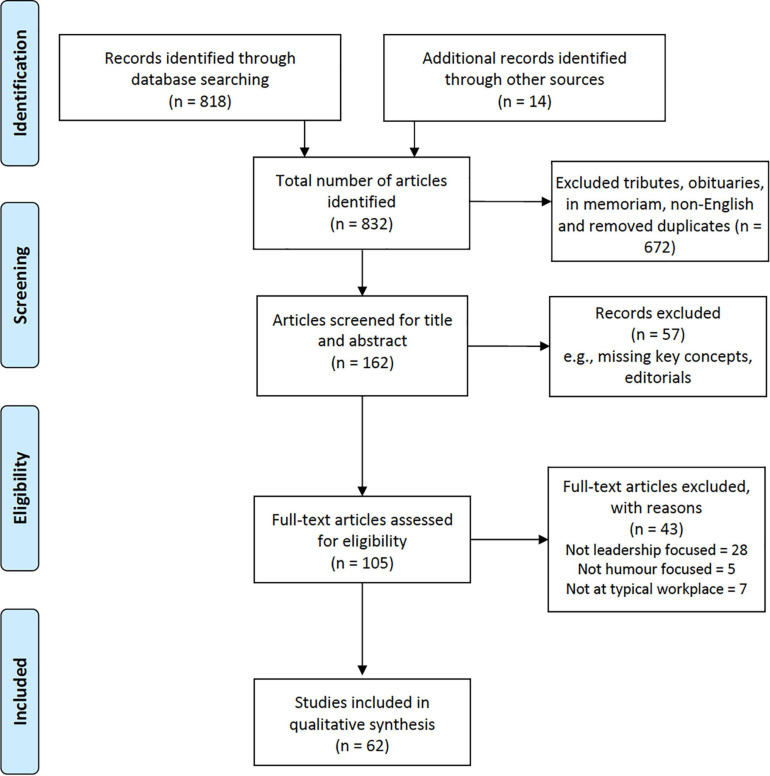
PRISMA flow diagram for searching and screening results.

The systematic search process identified 818 articles using the search strategy. The PRISMA diagram in Figure 1 illustrates the study screening and selection processes, resulting in the 62 studies included in the review.

### Step 4: Charting the Data

**FIGURE 2:**
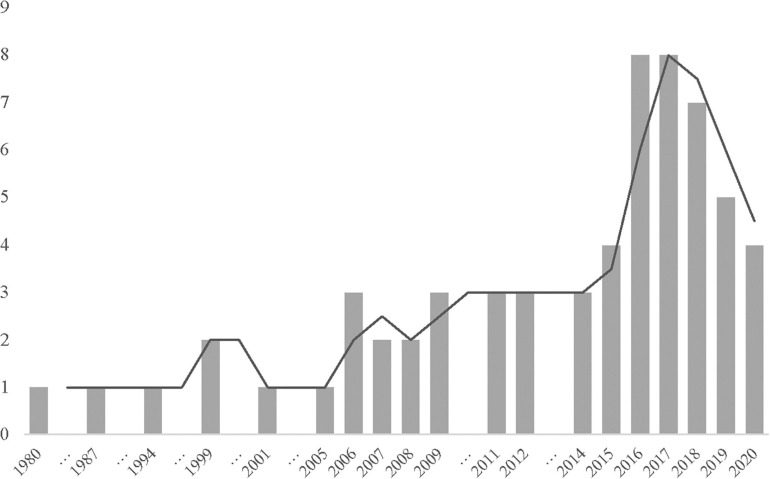
The number of peer-reviewed publications related to humor and workplace leadership. Trend line represents moving average. 2019 figure does not represent the full calendar year.

A data charting form (see Supplementary File) was developed to capture key information from the selected studies. Final data extracted from each study included: author, year of publication, study origin, purpose of study, participants, research type, and instruments used. There was also contextual information about the themes and the function or effect of humor for each study. The number of articles included each year is illustrated in Figure 2 and shows increased attention to the topic of humor and workplace leadership, particularly since 2016.

### Step 5: Collating, Summarizing, and Reporting Results

In order to identify the key themes of the studies included in this review, the data extracted and collated in the charting form were analyzed using thematic analysis. Following Braun and Clarke’s thematic analysis approach (Braun and Clarke, 2006), the coding process started with identifying the key concepts from the purpose of the study and reviewing the keywords of each study, the key concepts and keywords were grouped during the screening process, particularly abstract and full-text screenings. These groups of key concepts and keywords were then used to categorize studies by themes and subthemes. With this method, multiple themes may be coded to one study.

Three research aims were proposed in this scoping review that focused on identifying methodologies and guiding theoretical frameworks, reviewing and synthesizing the last 40 years of research and identifying key challenges, gaps, and making recommendations for future research directions. The following section reports on the findings of the scoping review and discusses the findings against each research aim.

## Results

### Overview of Methods and Theories

A summary of the 62 studies included in this review can be found in Supplementary File. Around 40% of the studies originated from the United States. The first study outside of North America was from New Zealand in 2006 (Holmes and Marra, 2006), and the first Australian study was published in 2017 (Wijewardena et al., 2017).

Of the studies included in this review, there was one meta-analysis focused on positive humor use in the workplace (Mesmer-Magnus et al., 2012), and one meta-analysis focused on leader trait humor vs. humor expression (Kong et al., 2019). As briefly mentioned in the introduction, the two reviews address different segments of this research area, though the overall research landscape remains uncharted.

Seven qualitative studies are included in this review, two of which used recordings from the Wellington Language in the Workplace Project (LWP) (Holmes and Marra, 2006; Schnurr, 2008). All seven qualitative studies used discourse analysis of meeting recordings and transcripts to demonstrate how humor can be used as a communication and discursive strategy to build rapport (Petraki and Ramayanti, 2018), construct leadership identity (Holmes and Marra, 2006; Välikangas and Tienari, 2018), and styles or to negotiate and establish power (Holmes and Marra, 2006; Rogerson-Revell, 2007, 2011; Schnurr, 2008; Watson and Drew, 2017). Six of the seven studies used conversation interactions between leaders and their followers, one study interviewed CEOs only (Välikangas and Tienari, 2018). There were 44 quantitative studies, of which 17 used the Humor Style Questionnaire (Martin et al., 2003) or part thereof, to assess leader humor use. Other humor measures used include the Multidimensional Sense of Humor Scale (MSHS) by Thorson and Powell (1993), and a five-item scale developed by Avolio et al. (1999) to assess leaders’ positive humor use frequency, based on previous work by Howell and Avolio (1988) and Dubinsky et al. (1995). The most common assessment approach of the quantitative studies in this review was to survey employees only in relation to leader humor (for example Pundt and Herrmann, 2015; Goswami et al., 2016). Twelve of the 44 quantitative studies used employee and leader dyadic pairs to assess leader humor (for example Avolio et al., 1999; Vecchio et al., 2009; Wisse and Rietzschel, 2014; Robert et al., 2016). Only one study investigated the effect of humor on leadership emergence, leadership as a process of influence (Watson and Drew, 2017). A majority of the quantitative studies adopted a cross-sectional approach, and three studies included experiential elements. The experiential interventions included cultural priming (Yue et al., 2016), temporary accessibility of participants’ moral identities (Yam et al., 2019), and manipulations of humor success or failure (Bitterly et al., 2017).

The theoretical basis of the humor studies included cognitive based theories: *incongruity theory*, *relief theory*, *benign violation theory*, and *superiority theory*. Several studies provided comprehensive summaries of these humor theories, for example Rogerson-Revell (2007) and Cooper (2008). Emotional based theories relating to humor were also used including *affective event theory* (AET) and *emotional contagion theory*. For example, Goswami et al. (2016) proposed that humor use leads to affective events, and according to AET these affective events trigger emotions and moods critical to workplace behaviors such as job attitude. C. D. Cooper et al. (2018) integrated three individual-resource-related theories: *social exchange theory*, *conservation of resources theory*, and *broaden-and-build theory* to argue that humor is a key interpersonal resource. However, Trif and Fodor (2019) used the Job Demands-Resources Model (JD-R) to demonstrate that aggressive humor in the workplace is a demand rather than a resource. The most referenced leadership theory (14.5%) was the *full range leadership model* consisting of: transformational, transactional, and laissez-faire leadership styles (Avolio et al., 1999; Avolio, 2011). The relationship-based theory *leader-member exchange* (LMX) was also adopted by several studies (Wisse and Rietzschel, 2014; Robert et al., 2016; Pundt and Venz, 2017; Wijewardena et al., 2017).

Four articles included in this review were not empirical studies but provided specific insights about humor in workplace leadership. For example, Vetter and Gockel (2016) presented the role of humor during organizational change, focused on three facets of the change process: coping with change; resisting the change; and leading the change. Five studies included in this review offered new theoretical propositions. For example, the *relational process model* by Cooper (2008) explains how humor operates to affect relationships in the workplace and the *wheel model of humor* by Robert and Wilbanks (2012) proposes that humor helps to initiate and perpetuate a cycle of individual and social-level positive affect. While both models have relevant components to workplace leadership, neither is specific for workplace leadership. The levels of analysis issue (Yammarino et al., 2005) is prevalent in the studies reviewed, theories that clearly define cross level relationships and model interactions are needed.

The overview demonstrated the studies on humor in leadership are heterogeneous and lack theoretical frameworks, which confirms the rationale for this scoping review. Investigating humor, as a phenomenon rooted in linguistics and discourse, qualitative approaches have especially relevant value (Litosseliti, 2018). The language used in workplace leadership may have unique characteristics compared with other contexts (Pondy, 1989; Seemiller and Murray, 2013), as such, a deductive approach with existing scales may not adequately detect the distinctiveness of humor used in a workplace leadership context. As a developing area of study, more qualitative exploratory studies, and narrative reviews that can synthesize the findings of these exploratory studies are needed. These studies will also facilitate theory development that are grounded in evidence.

### Key Challenges

#### Definitions

Scholars in humor studies tend to agree that humor has many facets, functions and styles (Martin et al., 2003; Romero and Cruthirds, 2006; Robert and Yan, 2007; Murata, 2014; Yang et al., 2017). In addition, various definitions of humor exist reflecting different academic perspectives (Wijewardena et al., 2017). Some view humor as a personality trait, describing it as “a way of looking at the world” (Thorson and Powell, 1993, p. 13). This results in the individual having a positive cheerful attitude or a habitual behavior pattern characterized by laughing frequently, and a tendency to joke with or amuse others (Martin et al., 2003; Mesmer-Magnus et al., 2012). Others view humor as a social phenomenon (Decker and Rotondo, 2001; Cooper, 2008; Robert and Wilbanks, 2012), a communication process shared between individuals (Lynch, 2002). From this perspective, humor is a skill that can be learned and developed until competent (Yang et al., 2017). Mesmer-Magnus et al. (2012) highlighted that researchers use the term “sense of humor” and “humor” interchangeably. Martin et al. (2003) summarized the conceptualization of “sense of humor” as a cognitive ability, an aesthetic response, a habitual behavior pattern, a trait, an attitude, coping strategy or defense mechanism. Most researchers are aligned in viewing “sense of humor” as “a personality trait that enables a person to recognize and use successful humor as a coping mechanism for social communication or interactions” (Mesmer-Magnus et al., 2012, p. 158).

Although these definitions encapsulate different aspects of humor, the utility of these definitions is limited, because they are a mixture of the dispositions, expressions, the functions and the operations of humor. These broad definitions cannot be operationalized into functional measuring instruments that facilitate quantitative investigations, nor can they sufficiently differentiate humor from similar concepts such as optimism or charisma.

In the workplace domain, there are also various definitions of humor. Romero and Cruthirds (2006, p. 59) defined humor as “amusing communications that produce positive emotions and cognitions in the individual, group, or organization.” Holmes and Marra (2006, p. 133) stated humor as “one valuable strategic resource in workplace discourse which leaders can choose to use where appropriate.” Wijewardena et al. (2017, p. 1318) used the term “managerial humor” and defined it as “any form of intentional and amusing communication, both formal and informal, that is created by the manager for the employee.” In proposing the *wheel model of humor*, Robert and Wilbanks (2012, p. 1072) defined a “humor event” as “discrete social behaviors that a producer intentionally creates for an audience that influences audience positive affect.”

These definitions are descriptive, characterized by conditions that may restrict the humor concept in workplace and leadership studies. For example, in the definition by Romero and Cruthirds (2006, p. 59) humor that produces negative emotions and cognitions is excluded; in Wijewardena et al. (2017, p. 1318) definition, unintentional humor is excluded; and the definition by Robert and Wilbanks (2012, p. 1072) excluded both negative and unintentional humor. These narrow definitions may be convenient for research, but could unwittingly skew the findings, leading to biased conclusions and potentially compounding the replication crisis in psychology (Lurquin and Miyake, 2017).

Some researchers do not offer a definition of humor but instead use observations to define the construct, particularly in qualitative studies (Cooper, 2008). For example, Rogerson-Revell (2011) used auditory and verbal cues, such as laughter, to identify humor episodes in the recordings of meetings used for data analysis.

The use of laughter in humor studies is problematic in distinguishing the effect of humor from the effect of mere laughter, as the two constructs are closely related but conceptually different. Also, laughter is not a reliable cue for humor, as it can be evoked by nervousness or embarrassment rather than humor (Ruch and Ekman, 2001). A definition of humor in workplace leadership studies should consider the unique workplace context and leader/member relationships. The definition also needs to be broad enough to capture the full range of humor types, styles and outcomes, so the effects of humor, both positive and negative can be examined in the workplace leadership context.

Similar to humor, leadership also has its challenges in definition (Day and Antonakis, 2012; Yukl, 2013), and how it differs from management (Kotterman, 2006; Antonakis and Day, 2017). For example, both leadership and management describe a position or a behavior, a manager can show leadership, and at times, leaders need to perform management tasks. Although theoretically, researchers or leadership professionals attempt to differentiate leadership and management (Kotterman, 2006), practically, the differences are rarely reinforced in empirical leadership studies. This is reflected in the authors’ observations of the recruitment of participants in leadership studies, which appear to be largely based on organizational positions, or self-identification of role, rather than a selection tool that differentiates leaders from managers. In other words, many of the empirical leadership studies are non-discriminant of leaders vs. managers.

#### Measurements

The diverse definitions of humor have contributed to the challenge of analysis. Martin et al. (2003) summarized the range of self-report measures that focus on certain aspects of humor. For example, the *Situational Humor Response Questionnaire (SHRQ)*, assesses the degree to which individuals smile and laugh in a wide variety of situations (Martin and Lefcourt, 1984); *the Coping Humor Scale (CHS)*, assesses how humor is used as a coping strategy (Martin, 1996); *the Sense of Humor Questionnaire (SHQ)* assesses how individuals notice and enjoy humor (Svebak, 1996). Martin et al. (2003) also developed the *Humor Styles Questionnaire* (HSQ), which assesses four dimensions relating to different uses or functions of humor. This measurement tool has been widely used in workplace humor studies as it provides a common framework for researchers to analyze the various organizational outcomes that are associated with the different types of humor (Pundt and Herrmann, 2015; Kim et al., 2016; Robert et al., 2016; Tremblay and Gibson, 2016).

Despite its popularity, the HSQ has similar issues to that which Martin criticized of the early measures. It is a measurement of *humor styles*, and categorized by its functions: affiliative, self-enhancing, aggressive and self-defeating. All the instruments, including SHRQ, CHS, and SHQ, are self-reporting measures without the necessary control for social desirability bias or unconscious biases stemming from different levels of self-awareness. Also, SHRQ, CHS, and SHQ are developed for the purpose of stress-buffering effects of humor, one of many potential outcomes of humor. Therefore, in order to further the research of humor in leadership, instruments that are fit for purpose need to be developed, specifically for the context of workplace leadership.

The measures for leadership are predominantly at individual and dyadic levels. Similar to the proxy construct used for humor, leadership is often operationalized as different leadership styles, for example, transformational leadership (Avolio et al., 1999; Goswami et al., 2016), ethical leadership (Valle et al., 2018). Leader-Member Exchange (LMX) is used to measure dyadic relationship (Pundt and Herrmann, 2015; Wijewardena et al., 2017).

### Key Themes

Based on the research focus of the studies included in this review, this section reports and discuss the key themes identified. The research synthesis using Braun and Clarke’s (2006) thematic analysis approach identified four key themes: (1) the effect of humor style on individual and organizational outcomes; (2) humor as a communication tool and discursive resource; (3) the moderator and boundary conditions of effective humor use by leaders; and (4) cultural influence on humor perception and experience. The four key themes and relevant sub-themes are shown in Table 4 and outlined in detail below.

**TABLE 4:** Summary of themes and subthemes.

Theme	Subthemes
Humor styles and outcomes	Effect on individual outcomes (e.g., attitude, satisfaction, stress-buffering, positive emotions, psychological capital and wellbeing)
	Effect on leadership outcomes (e.g., LMX, power, trust, perception of leader effectiveness)
	Effect on Organizational outcomes (e.g., Employee engagement and performance, citizenship behaviors, creative and innovative, and inclusive culture)
Humor as a communication tool and discursive resource	To create team, by establishing solidarity and cohesion of a group
	To control power distance between leader and follower
	To “save face,” minimize the impact of negative messages
	To relax atmosphere and cultivate creativity and innovation
Variabilities in humor and leadership relationship	Trust
	Gender
	Appropriateness
	Leadership styles
	Personal preferences
Cultural context	Cross-cultural differences
	Intercultural factors

####  Humor Styles and Outcomes

**FIGURE 3:**
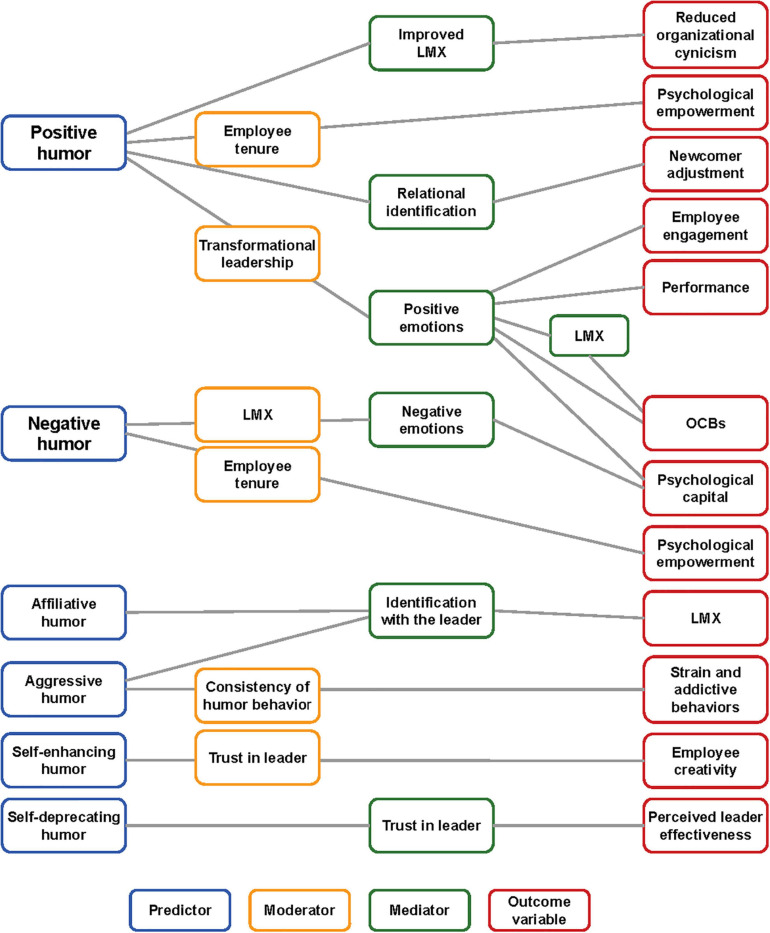
An illustrated summary of the outcome variables of the studies that investigated different humor styles.

There was general agreement across the studies that humor is a complex construct with many dimensions and is therefore difficult to define (Martin et al., 2003; Robert and Yan, 2007; Mesmer-Magnus et al., 2012). Humor styles, however, could be more objectively determined based on the functions or outcomes of the humor experience, therefore providing an easier construct for researchers to assess in the quantitative studies. The framework most commonly used was the one developed by Martin et al. (2003). This framework initially categorizes individual humor use by assessing the extent to which humor use is adaptive or maladaptive to wellbeing. Two categories based on the target direction of the humor use, either inwardly or interpersonally, are then differentiated into four humor types: (1) the interpersonal adaptive style (affiliative humor); (2) the inwardly adaptive style (self-enhancing humor); (3) the interpersonal maladaptive style (aggressive humor); and 4) the inwardly maladaptive style (self-defeating humor) (Martin et al., 2003). Figure 3 provides an illustrated summary of the outcome variables of the 9 studies reviewed that investigated different humor styles.

Adaptive or positive humor styles appear to have attracted greater attention from researchers perhaps because they are associated with desirable workplace outcomes. The key findings of studies related to positive humor use were summarized in the meta-analysis by Mesmer-Magnus et al. (2012). An examination of the 49 independent studies (*n* = 8,532) in this meta-analysis found that positive humor use by leaders was associated with enhanced work performance (Vecchio et al., 2009), greater work satisfaction of subordinates (Hughes and Avey, 2009), better workgroup cohesion (Fine and De Soucey, 2005), and a positive perception of leader and satisfaction with leader performance (Decker and Rotondo, 2001). Additional findings in relation to positive leader humor use and workplace outcomes identified after the meta-analysis include:

• Improved leader member relationship based on the theory of Leader—Member Exchange (LMX) (Pundt and Herrmann, 2015; Robert et al., 2016; Petraki and Ramayanti, 2018).
• Improved employee core self-evaluation and trust in leaders (Karakowsky et al., 2020; Neves and Karagonlar, 2020).
• Reduced social and power distance and status disparities (Bitterly et al., 2017).
• Improved employee psychological capital and wellbeing (Kim et al., 2016; Wijewardena et al., 2017).
• Elevated work engagement, satisfaction, performance and Organizational Citizenship Behaviors (Decker, 1987; Goswami et al., 2016; de Souza et al., 2019; Guenzi et al., 2019; Mills et al., 2019; Neves and Karagonlar, 2020).
• Enhanced ability to manage organizational change (Vetter and Gockel, 2016).
• Increased levels of innovation and creativity (Hughes, 2009; Lee, 2015; Pundt, 2015; Salas-Vallina et al., 2018).
• Newcomer adjustment (Gkorezis et al., 2016).

**FIGURE 4:**
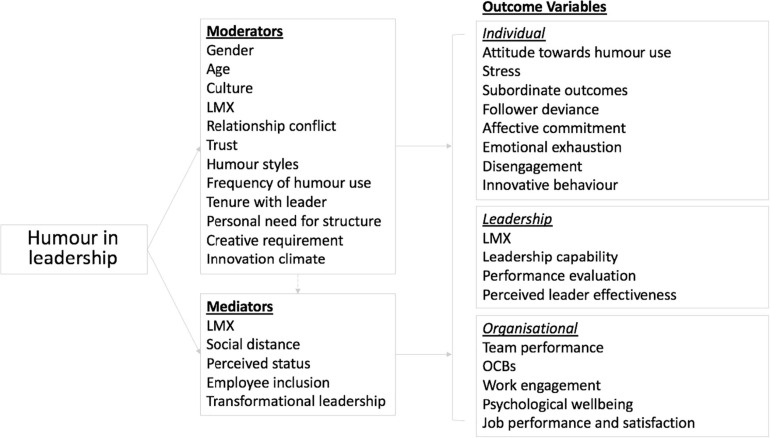
An illustrated summary of the effects of humor used in leadership in terms of individual, leadership, and organizational outcomes with associated moderators and mediators.

Three studies investigated negative leader humor style, specifically aggressive humor. One studied the impact of aggressive humor on employee wellbeing, in terms of strain and addictive behaviors (Huo et al., 2012); another explored its effect on employees’ intention to leave. The third study examined the moderating effect of aggressive humor on the relationship between abusive supervision and dysfunctional resistance, where the relationship was weak at low levels of aggressive humor and stronger at high levels of aggressive humor (Goswami et al., 2015). Malone (1980) postulated the case for and against humor in the workplace with a series of questions mostly focused on appropriate/inappropriate use of humor and positive/negative reactions to humor use. More recently researchers have begun to examine the mixed effect of humor style. For example, Wisse and Rietzschel (2014) and Pundt and Herrmann (2015) studied the opposing effects of affiliative and aggressive humor in leadership and the relationship with LMX. Yam et al. (2018) integrated benign violation theory (McGraw and Warren, 2010) and social information processing theory (Salancik and Pfeffer, 1978) and proposed that although a leader’s humor is positively associated with LMX and work engagement, it can also foster followers’ deviance by signaling the acceptance of norm violation at work. Figure 4 provides an illustrated summary of the effects of humor used in leadership in terms of individual, leadership, and organizational outcomes from the 44 quantitative studies reviewed, regardless of humor style.

####  Humor as a Communication Tool and Discursive Resource

Discursive studies of naturally occurring interactions at work involving humor appear to be the most direct type of analysis undertaken regarding workplace leadership and humor. Communication is also one of the fundamental and crucial aspects of leadership effectiveness. Bligh and Hess (2007) noted that leadership is fundamentally grounded in language and rooted in communication processes. As previously discussed, humor is difficult to assess quantitatively due to its subjectivity and fluidity in definition. Qualitative research, however, can avoid these challenges. The findings of the five qualitative studies included in this review were similar in terms of how leaders use humor in a discursive manner. This includes:

• To *create team* by establishing solidarity and cohesion of a group (Holmes and Marra, 2006; Rogerson-Revell, 2007; Watson and Drew, 2017) or a sub-group (Schnurr, 2008). This strategy can be advantageous to the in-group members who mirror each other’s behavior; but detrimental to the out-group if some members do not subscribe to the in-group behaviors (Rogerson-Revell, 2007).
• To *do power* by establishing authority, assertiveness and leadership identity to get things done (Schnurr, 2008; Rogerson-Revell, 2011; Watson and Drew, 2017), or maintaining a hierarchical relationship and reinforcing power boundaries (Holmes and Marra, 2006). An alternative is to minimize status difference and downplay one’s authority in order to cultivate relationally oriented behaviors and a positive work atmosphere (Schnurr, 2008).
• To *be polite* as described by Brown and Levinson (1978), in terms of a positive politeness strategy. Despite being criticized as an oversimplified view of humor (Rogerson-Revell, 2007), this strategy highlights the function of humor in protecting one’s dignity and resolving tension (Schnurr, 2008), softening the impact of negative messages (Holmes and Marra, 2006), and preserving the group’s “collective face” (Watson and Drew, 2017).
• To *generate energy* by contributing to humorous events and encouraging “bursts of creative mental and intellectual activities” (Holmes and Marra, 2006, p. 132) for innovation and problem solving (Holmes and Marra, 2006, p. 132; Rogerson-Revell, 2011).

#### Variability in the Humor and Leadership Relationship

Based on the literature, there is little doubt that a relationship between leader humor use and leadership outcomes exists; however, the characteristics of the relationship cannot be easily identified. The challenges stem from both the difficulty regarding definition and measurement of humor as well as the context of the leadership situation. As a result, any factors associated with leadership or workplace characteristics can potentially become a moderator of the relationship between humor and leadership outcomes. These mediators/moderators are illustrated in Figures 3, 4 and include trust in the leader, leader gender, and situational factors affecting the appropriateness of humor.

#### Trust

Three of the studies included in this review used trust as a moderator of humor use and organizational outcomes. Lee (2015) demonstrated that the relationship between self-enhancing humor and employee creativity became stronger as trust in the leader increased. Kim et al. (2016) found the relationship between affiliative humor and social distance was stronger when trust in leaders was high. Trust was also reported by Tremblay (2017) as a boundary condition regarding the effectiveness of a humor climate in the workplace, whereby leader humor effects employee inclusion and humor climate. Trust has also been used to explain the relationship between leader self-deprecating humor and perceived effectiveness. A mediating effect was analyzed by Gkorezis and Bellou (2016). They found that leader self-deprecating humor, specifically willingness to make humorous comments about personal weaknesses, was perceived as transparent communication and was more likely to yield trust from followers and indirectly influence followers’ perceptions of leader effectiveness.

#### Gender

Decker and Rotondo (2001) proposed the humor, manager gender, and leader behavior and effectiveness model. The authors found two moderating effects of gender based on humor style. Firstly, despite female leaders using less positive humor, there was a stronger relationship between female positive humor use and perceived leader behavior and effectiveness compared with male leaders. Secondly, when negative humor was used, the moderating effect of gender was the opposite in that the overall negative effect was less for male compared with female leaders. Vecchio et al. (2009) could not replicate the gender moderating effect, however, and argued that the failure to obtain statistical significance might have been a function of the statistical power of their study. A cross-cultural study by Decker et al. (2011) found the moderating effects of gender to be in the opposite direction with a Chinese sample, compared with the earlier findings by Decker and Rotondo (2001) in a Western context. In particular, male leaders benefited more than female leaders from positive humor and were harmed more by negative humor. Social expectations in China were used to explain the findings. In China, male leaders are perceived as more serious than females in business settings, so their effort in using positive humor may be especially appreciated by followers; negative humor portrays male leaders as even more unapproachable (Decker et al., 2011). Evans et al. (2019) argued that gender stereotypes moderate how humor is perceived, based on the parallel-constraint-satisfaction theory. The interpretation of observed humor is not only based on the humor itself, but also the evaluation of behavioral deviation against our mental model for each gender.

#### Appropriateness

Reference to humor appropriateness in the literature is generally synonymous with humor style, with positive humor styles being appropriate and negative humor styles inappropriate (Hughes, 2009; Bitterly et al., 2017). Bitterly et al. (2017) attempted to investigate the moderating role of appropriateness in the relationship between humor and perceived competence. The study found that even though all humor use projects confidence, inappropriate humor signals a lack of competence and can decrease leader status.

#### Other Variables

**FIGURE 5:**
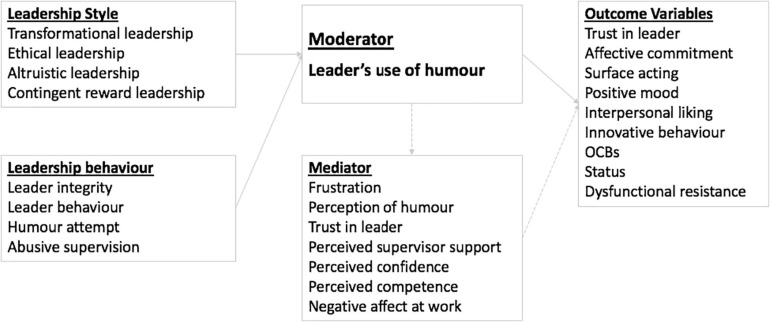
An illustrated summary of the studies that investigated interactions between leadership style, behavior and leaders’ use of humor, and their potential outcomes (directly or indirectly) via mediating variables.

Leadership styles and behaviors, in particular transformational leadership strengthens the relationship between leader positive humor and followers’ positive emotions at work (Goswami et al., 2016). The tenure the employee has with a manager also moderates the relationship between the leader’s humor use and the employee’s psychological empowerment (Gkorezis et al., 2011). Humor can also moderate the relationship between leadership style and performance (Avolio et al., 1999) as well as follower attitudes, such as trust, identification, affective commitment, job satisfaction or frustration (Hughes and Avey, 2009; Valle et al., 2018). Figure 5 provides an illustrated summary of the ten studies that investigated interactions between leadership style, behavior and leaders’ use of humor, and their potential outcomes (directly or indirectly) via mediating variables.

Frequency of humor has been found to have a positive impact in the workplace. Hurren (2006) found that teachers experience higher job satisfaction when principals frequently engage in humor at work. Boundary conditions relating to humor use have also been examined. Pundt and Venz (2017) found that followers with a strong preference for hierarchical and clear social structures, reacted less positively to humor from leaders and that commitment and engagement was weaker for followers with a high personal need for structure.

Humor in leadership, and its various forms and constructs were examined in the studies as a predictor, a moderator, and a mediator, however, the antecedents of humor in leadership remain absent in the current literature.

#### Cultural Context

The role of culture has been identified as one to the key factors in humor studies. Eight papers included in this review were explicitly focused on how culture influences the relationship between leaders’ humor use and leadership outcomes. The contexts of these studies were not universal. Six of the eight studies focused on cross-cultural differences between Eastern and Western cultures. These studies compared and contrasted Eastern cultures (typically represented by China) and Western cultures (typically represented by the US or Australia) in terms of how people perceive humor and how leaders use humor in their own native cultural environment (e.g., Wang et al., 2017; Yang et al., 2017). One study investigated intercultural variability, whereby participants from different cultural backgrounds interact in the same workplace (Rogerson-Revell, 2007) and one study offered theoretical propositions related to intercultural and cross-cultural studies (Robert, 2005).

Differences highlighted in the cross-cultural humor studies were mainly based on Hofstede (1980)’s *cultural dimensions theory*, in particular, power distance and individualism vs. collectivism. Humor is perceived to be more acceptable and effective in low power distance and individualistic cultures compared with high power distance and collectivistic cultures (Robert, 2005; Wang et al., 2017). In Western cultures, humor is perceived to be a positive and desirable leadership quality, whereas in Eastern cultures humor is associated with *intellectual shallowness* (Yue et al., 2016; Yang et al., 2017) and “a natural antithesis between reverence and humor” (Decker et al., 2011, p. 44). Decker et al. (2011) found that negative humor was not considered as humorous in China and was negatively associated with ratings about task and relationship leadership styles. The traditional Chinese view of “there’s no humor in misfortune, and humor is only important for professional entertainers with special expertise and talent” could explain this finding. Confucian doctrines, such as “a man has to be serious to be respected” (Yue et al., 2016, p. 1495) could also account for these perceptions about humor in Eastern cultures.

As globalization spreads, organizations require collaboration of people from different cultural backgrounds. This trend highlights the importance of intercultural research, in comparison to cross-cultural research. This is especially critical for countries where populations are characterized as “multicultural” with a high proportion of migrants. Rogerson-Revell (2007) found that when people attend international business events, they usually adjust their humor style to suit the interactive context. The study by Rogerson-Revell (2007) found considerable differences in the use of humor between individuals within meetings, and within individuals between meetings. This suggests a multi-layered relationship between humor use and situational factors such as the role the attendees are appointed to (e.g., the expert or the chair of the meeting) and/or the dominant interactive style at the event (e.g., formal or informal).

## Discussion

This scoping review included a wide range of studies on the topic “humor in workplace leadership” from the last four decades. Through a systematic search of the literature, four prevalent themes of studies were identified. The first were studies using humor styles as the proxy construct to humor, examining the effect on different levels of outcome variables (individual, leadership and organizational). The second theme included studies focused on humor as a communication tool and discursive resource, used to achieve relational goals. Third were studies that investigated moderators and boundary conditions of the effect of humor on leadership. Last were studies investigating cultural influences on humor perception and experience. Grouping the empirical research from the last 40 years into these key themes addressed the first aim of this scoping review. In the following sections, we discuss and address the second and third aims—mapping the progress and gaps that still persist, and making recommendations for future research directions including specific research questions to guide future systematic reviews. We also provide a summary of theoretical and practical implications of this review, note the limitations and offer concluding remarks.

### Research Progress and Gaps From the Last 40 Years

**FIGURE 6:**
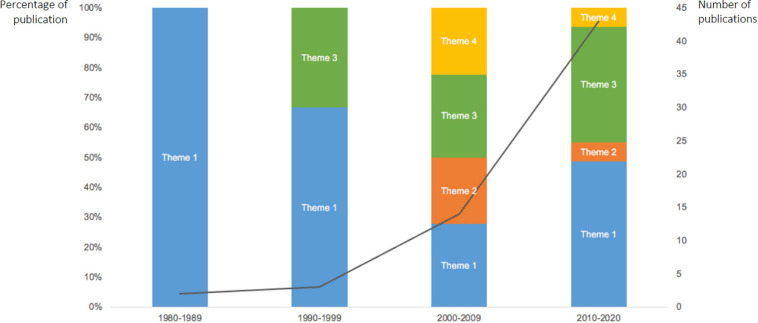
An illustrated summary of the shifts in research interests across the last four decades. Theme 1, humor styles and effects; Theme 2, humor as a communication tool and discursive resource; Theme 3, variables in the humor and leadership relationship; Theme 4, cultural context. The trend line represents the number of publications of each decade.

The themes identified in this scoping review capture the key focus of the research interests across the last 40 years. We undertook a mapping exercise to identify how the research interests in relation to this topic have shifted across the four decades. Figure 6 provides a visual illustration of the identified patterns. Following is a commentary against these patterns to elucidate the research interest trajectories based on the published studies included in this review. The discussion in the subsequent section considers the progress and gaps on the topic of humor in workplace leadership more broadly, guided by the research questions raised in the field by Malone (1980). The aim is to demonstrate the evidence and rationale for future research recommendations.

#### The Shifts and Trajectories of Research Interests in the Current Body of Work

Four key patterns emerged in analyzing the shifts and trajectories of the research interests. Firstly, early research (before the new millennia) was mainly focused on theorizing the effects of humor use and testing the hypothesized effects quantitatively (Theme 1). The frequency of studies within the first two decades was minimal, but toward the end of this period, researchers began to investigate humor as a moderator of the relationship between leadership styles and leadership effectiveness (Theme 3). Secondly, Themes 1, 3 continued to dominate research interests into the 2000s. This was particularly so after 2005, and 2 years after the publication of Martin et al.’s (2003) Humor Styles Questionnaire (2003), which enabled standardized measurements of humor, and triggered an increase in quantitative studies, including cross-cultural and inter-cultural comparison projects (Theme 4). Thirdly, qualitative studies began to emerge in the mid-2000s, focused on humor as a discursive resource and communication tool (Theme 2). Qualitative research increased slightly into the 2010s, however, the percentage reduced because of the number of studies focused on the other themes. Finally, the complexity of research has increased exponentially over time, especially in the last decade. This is reflected by the number of research themes identified in these studies. Research pre-2010 is generally represented by one of the four themes, whilst studies post-2010 tend to include 2 or 3 of the themes. For example, in their 2017 study, Yang et al. investigated the moderating effect of interaction formality on the relationship between humor and leadership (Theme 3), and the cultural differences in attitudes toward humor (Theme 4). Complexity is also reflected through the sophistication of research designs. There were more longitudinal studies and studies investigating multi-level effects, (i.e., individual level as well as group level effects).

While it is encouraging to see the progress made and the patterns of research development over the last four decades, it is also critical to review the current body of work against the practical use of humor in the working environment and leadership context. The following questions, originally proposed by Malone (1980) are used to guide this review relating to progress and research gaps.

#### Can Humor, Properly Used, Serve as a Tool to Enhance the Leadership Process?

This review indicates that the extant research does support the proposition that humor can serve as a tool to enhance leadership effectiveness, if used appropriately. However, what constitutes “appropriate use” is yet to be determined. To date, the humor styles framework based on the motivations of humor use (Martin et al., 2003), is the only current method of assessing appropriate use. Dimensions other than humor style should also be explored, such as the characteristics of the situation and the authenticity of leaders’ use of humor.

#### Can Humor Be Used Effectively by Most Leaders or Should the Use of Humor Be Reserved to Those Who Are Naturally Funny?

The fundamental challenge of this question is whether humor can be viewed as a capability or trait. Some researchers have described humor as an individual trait that differentiates individuals with the propensity to create and appreciate humor from others (Thorson and Powell, 1993). However, much of the research in management views humor as a social phenomenon, an intentional shared event with the purpose of amusing others (Cooper, 2005, 2008). From a trait theory perspective, an individual’s propensity for and appreciation of humor use is unlikely to change, that is, leaders either can or cannot use humor effectively. As such, perhaps humor should be reserved for those who are naturally funny. However, from a social phenomenon perspective, if use of humor is a social skill, then this question can be addressed quite differently and like other leadership skills, could be cultivated and developed through training. The recent review by Kong et al. (2019) serves as a strong guiding post in this area, their meta-analysis indicated that leaders’ humor behavior or expression has stronger influence on followers’ outcomes in comparison with leaders’ trait humor. The research question then changes from “Can humor be used effectively by most leaders?” to “How can humor be used effectively by most leaders?” and directs future research toward training leaders in how to effectively use humor as part of leadership development programs. It may not be a choice between the two perspectives, rather a combination of both, but it has not been investigated to date. The opportunities of development are not limited to training the leaders to be more spontaneous with humor, but also recognize humor residing in the relationship with others. The leaders may or may not be naturally humorous, but developing sufficient sensitivity to the situational cues and signals from others is an opportunity for leaders to use humor effectively.

#### Under What Conditions can Humor Be Used Most Effectively; Under What Conditions Is Humor Inappropriate?

From an interactionist point of view, behavior is a function of both the person and the situation (Mischel, 1979; Blum et al., 2018). In order to fully understand behavior, in this case use of humor as a leadership capability, the characteristics of the person and the characteristics of the situation need to be analyzed and understood (Decker and Rotondo, 1999). The *strong situation hypothesis* (Cooper and Withey, 2009) may be a suitable framework for analyzing the characteristics of situations where humor can be used most effectively. It is recognized that some situations constrain behavior variabilities, so most people will show the same level of behavior in these situations, which are known as *strong* situations; and by contrast, in *weak* situations, people enact different levels of behavior. The strong situation hypothesis suggests personality matters most in weak situations and least in strong situations. If the “strong situation” characteristics for humor use can be described and controlled, then the differentiation between the trait and the skill components of humor can be more systematically delineated. This understanding could assist researchers and scholars in integrating humor as a trait and a social skill. Although, not related to the competencies in humor production, awareness of the characteristics of a “strong situation” for humor use could help leaders develop sensitivity to situational cues and signals, more commonly known as the ability to “read the room,” potentially resulting in more appropriate use of humor in leadership. For example, many leaders may intuitively understand that the opening speech at an end of year work gathering is a “strong situation” for “spontaneous” humor; and that performance review meetings are “weak situations” for humor, especially humor that is personal or negative. If a systematic approach for assessing situations for humor use was developed, it may assist leaders who are less intuitive about humor use.

#### What Types of People Respond Most Readily to Humor, What Types of People Are Most Likely to React Negatively?

There is limited evidence addressing this question about the types of people that respond positively or negatively to humor. The research related to boundary conditions of humor can shed some light. For example, Pundt and Venz (2017) found followers who strongly prefer hierarchical and clear social structures, react less positively to humor in leadership. Bitterly et al. (2017) found individuals’ confidence and competence were two dimensions associated with effective humor. The appropriate use of humor signals both confidence and competence of the instigator, while inappropriate use of humor may still project confidence, but suggests low competence. Investigating the converse relationship would be interesting (i.e., does an individual’s confidence influence humor use propensity?) One of the qualitative studies included in this review found that Chairs of meetings are often the instigators of humorous events (Rogerson-Revell, 2011). There are currently no personality-based studies to differentiate how people at work respond to leader humor use (positively or negatively). Studies outside of the workplace leadership domain may exist but they were excluded from this review.

#### What Types of Humor Are Most Effective; What Types Are Most Likely to Produce Negative Reactions?

Humor types and styles have been adequately addressed by researchers. Using the well referenced Humor Styles Questionnaire (Martin et al., 2003), several studies demonstrated that positive humor styles, (affiliative and self-enhancing humor) are the most effective while negative humor styles, (aggressive and self-defeating humor) are the most likely to produce negative reactions (Martin et al., 2003). Researchers have made significant progress in understanding the differing effect that positive and negative humor styles have on individual, organizational and leadership outcomes. Moderating factors and boundary conditions have also been studied in different cultural contexts.

### Recommendations for Future Research Directions

The challenges associated with the lack of an agreed definition and the need for theory and instrument development, should continue to be at the forefront of future research, especially in the context of workplace leadership studies. In addition, the research gaps identified in the section above indicate the future research directions that are valuable to both academics and leadership professionals alike. The following research questions highlight key opportunities where empirical studies can add value to the body of knowledge, and the focus of theory development for the study of humor in leadership, as well as serving as potential review questions for future systematic reviews in this field of study.

• *What constitutes “properly used” humor, or humor appropriateness in leadership?*

Perhaps this will guide researchers toward a functional definition of humor in workplace leadership and enable the development of a measurement instrument that has better utility than styles alone.

• *What conditions influence the effectiveness of humor in leadership?*

Acknowledging both humor and leadership occur at individual, group and organizational levels, future research should address the levels of analysis issue by developing theories and models that incorporate cross-level conditions and interactions. The conditions of influence can also occur at different levels. For example, as illustrated through this review, gender or preference at individual level; trust or conflict at dyadic level; work climate at the organizational level; culture or social structure at the societal level. The clear identification of these conditions will help leaders and leadership teams to better utilize humor as a discursive tool, harnessing the positive effects while navigating through the dangerous terrain in the workplace. Only when the situational conditions of effective humor use are identified, researchers can start to delineate the effects of the person vs. the situation as outlined in the strong situation hypothesis (W. H. Cooper and Withey, 2009).

• *How do trait and personality theories interact with humor and leadership theories?*

Both humor and leadership have trait and behavioral components. Instead of comparing which component has better predictive power of workplace outcomes, understanding the mechanisms and commonalities of the trait component between humor and leadership, or the triad relationships among trait, humor behavior and leadership behavior, will add much more value in our understanding of humor in leadership. And this will inform the feasibility of the following research question.

• *Can humor appreciation and expression be learned, if so, how can they be most effectively developed?*

Although the studies that may answer this question are beyond the selection criteria of this review, and the question is not exclusive to workplace leadership, it is nevertheless relevant to the future direction of humor in leadership. If humor can be learned, perhaps like any other form of arts, through extensive periods of exposure and practice, then the researchers can focus on the latter part of the question, to find the most effective way of learning it. If humor cannot be learned, or cannot be learned authentically, and it is still desired, then the challenge for leadership scholars shifts from development of leaders to recruitment and selection of leaders who have this trait. Regardless of development or selection, being able to define and measure humor in leadership remains critical to future research.

• *What leadership skills and competencies or general skills facilitate effective humor use in workplace leadership?*

Related to the previous question, if humor can be learned and developed, what are the antecedents, mediating and moderating factors? Would leadership skills such as emotional intelligence, risk management, judgment and decision-making play a role in our ability to acquire humor as a skill? Or would general skills such as adult learning and theory of mind have more influence on the development? Can certain personality types learn humor easier than others? While these questions are intriguing, they may suggest more complex relationships, cyclical or reciprocal, among trait, skills, humor and leadership.

The above questions and research directions are not exhaustive by any means, but they represent the necessary next steps in understanding humor in leadership in a systematic and comprehensive way. To summarize the contribution of this review, we offer the following theoretical and practical implications.

### Theoretical Implications

The framework of humor styles developed by Martin et al. (2003) has contributed significantly to our understanding of the differences in humor use and the impact on individual, relational and organizational outcomes. However, as the complexity of humor in leadership studies grow, to advance humor research in the discipline of leadership, it demands new theories and frameworks that are specific to the context. This review has offered guidance in developing future humor in leadership theories and frameworks. In summary, the theoretical development needs to leverage knowledge from multidisciplinary areas such as emotional intelligence, personality and trait, and effective communication skills to understand humor use as a construct that has multiple latent elements. Also, tapping into learning theories will be fruitful in understanding the most effective way of acquiring skills related to humor use. Based on the Strong Situation Hypothesis, being in a leadership role creates specific demands on leader behaviors; humor use, therefore, can be interpreted as a response to that specific situational demand. Further theoretical development in this domain will help leaders understand the salient situational cues when using humor.

### Practical Implications

The most significant practical implication of this review is the understanding of humor beyond different humor styles. This scoping review informs leaders and scholars about the diverse range of humor functions; insight about why humor is “a double-edged sword”; the difference between trait and behavioral humor. This review also establishes the need for leaders to understand competency areas related to appropriate humor use in the leadership context. Future research that can delineate these competency areas will be critical in shedding light on how humor can be learned and used intelligently by leaders.

### Limitations

The inclusion of gray literature in this review may have provided a more comprehensive overview of the humor and leadership landscape (Levac et al., 2010), as well as providing anecdotes or common beliefs people hold about humor in workplace leadership. However, the academic intent of this scoping review, the diverse forms of gray literature and the vast amount of material available online, meant the feasibility of the study needed to be prioritized (Peters et al., 2015). In addition, the literature search concluded in May 2020 using four purposefully selected databases. The databases chosen reflected Psychology and Business, databases most likely to meet the research needs and search criteria. As a result, research published post May 2020 or not listed in any of the selected databases are thus excluded from this review.

## Conclusion

This scoping review identified the focus of existing research, the range of methodologies adopted, and guiding theoretical frameworks or propositions. Key findings relating to the function and effect of humor use, individual and organizational leadership outcomes regarding humor use, and the significant variables that influence the relationship between leaders and followers when humor is used were identified and synthesized. Future studies addressing these unanswered questions will advance and broaden leadership and leadership development studies, most specifically “Can humor be learned and effectively used by leaders?” and “under what conditions, (including personality type and situational context), can humor be used most effectively?” The reason that these two questions have not attracted any research to date could relate to the common belief that humor is innate and spontaneous, regardless of the situational context. Researchers could also consider theories and models available from other disciplines to identify the most effective way of developing humor in leadership. Studies in these areas would enable leaders to better understand and utilize humor and potentially achieve more effective leadership outcomes. This scoping review adds value to the scientific community by (1) synthesizing the research progress to date and current knowledge gaps for the body of work accumulated over the last 40 years; (2) outlining current research interests; (3) making recommendations about future directions; and (4) highlighting the challenges and opportunities for future scholars who wish to advance understanding and apply humor in the leadership context.

## Author Contributions

CR carried out the review procedures under the direct supervision of AW. CR took the lead in writing the manuscript. AW was the main editor of the manuscript. All authors provided critical feedback and helped shape the themes of the review, discussions and future research directions, conceived, and planned the review together.

## Conflict of Interest

The authors declare that the research was conducted in the absence of any commercial or financial relationships that could be construed as a potential conflict of interest.

## Publisher’s Note

All claims expressed in this article are solely those of the authors and do not necessarily represent those of their affiliated organizations, or those of the publisher, the editors and the reviewers. Any product that may be evaluated in this article, or claim that may be made by its manufacturer, is not guaranteed or endorsed by the publisher.
